# Advanced assessment of cardiac morphology and prediction of gene carriage by CMR in hypertrophic cardiomyopathy - the HCMNet/UCL collaboration

**DOI:** 10.1186/1532-429X-16-S1-O30

**Published:** 2014-01-16

**Authors:** Gaby Captur, Timothy J Mohun, Gherardo Finocchiaro, Robert Wilson, Jonathan Levine, Lauren Conner, Luis Lopes, Vimal Patel, Daniel Sado, Chunming Li, Paul Bassett, Anna S Herrey, Maite T Tome Esteban, William J McKenna, Christine E Seidman, Vivek Muthurangu, David Bluemke, Carolyn Y Ho, Perry M Elliott, James Moon

**Affiliations:** 1 Cardiac MRI Unit, The Heart Hospital, London, UK; 2 Department of Developmental Biology, MRC National Institutes for Medical Research, Mill Hill, UK; 3 Cardiovascular Genetics Center, Brigham and Women's Hospital, Boston, Massachusetts, USA; 4 Institute of Cardiovascular Science, University College London, London, UK; 5 Department of Radiology, University of Pennsylvania, Philadelphia, Pennsylvania, USA; 6 Biostatistics Joint Research Office, University College London, London, UK; 7 Department of Genetics, Harvard Medical School, Boston, Massachusetts, USA; 8 UCL Centre for Cardiovascular Imaging and Great Ormond Street Hospital for Children, Great Ormond Street Hospital for Children, London, UK; 9 Radiology and Imaging Sciences, National Institutes of Health/Clinical Center, Bethesda, Maryland, USA; 10 The Inherited Cardiovascular Disease Unit, The Heart Hospital, London, UK; 11 Howard Hughes Medical Institute and the Cardiovascular Division, Brigham and Women's Hospital, Boston, Massachusetts, USA

## Background

Myocardial architectural abnormalities, have been identified in hypertrophic cardiomyopathy(HCM) gene mutation carriers without hypertrophy(G+LVH-). Some of these changes may be related to the underlying mutation, but whether they can predict gene carriage in relatives of HCM probands is unknown. Cardiac trabeculae may be prominent in overt HCM, suggesting they could form part of this constellation of abnormalities but previous techniques have not permitted more detailed study. We developed a fractal method for quantitation of trabeculae, tracked their development in embryonic mice and applied it to humans imaged by CMR. We hypothesize that fractal analysis may detect abnormal trabeculae in HCM mutation carriers before development of LVH and that a combination of cardiac architectural abnormalities could be used to predict gene carriage in HCM.

## Methods

TRABECULAE IN MOUSE EMBRYONIC DEVELOPMENT-63 Murine hearts were examined from the time of ventricular septation(E14.5) till just before birth(E18.5). Trabeculae ware charted by fractal analysis of high-resolution episcopic microscopy images using a box-counting method. HUMAN MORPHOLOGY-74 G+LVH- sarcomere mutation carriers(29 ± 13 yr[SD]|51%M) were identified in 12 US-centers(HCMNet|n = 35) and UCL(n = 39). Subjects underwent CMR and fractal analysis. Results were compared with 111 overt HCM patients(G+LVH+|n = 71;G-LVH+|n = 40) and 136 matched controls(36 ± 16 yr|63%M). We analyzed a single-center(UCL) G+LVH- case-control cohort to identify factors associated with gene carriage, evaluating anterior mitral valve leaflets(AMVL), wall thickness, clefts, trabeculae and other variables. We validated identified associations in the multi-center HCMNet cohort, and combined significant parameters into a model for predicting genetic carriage.

## Results

**Figure 1:**
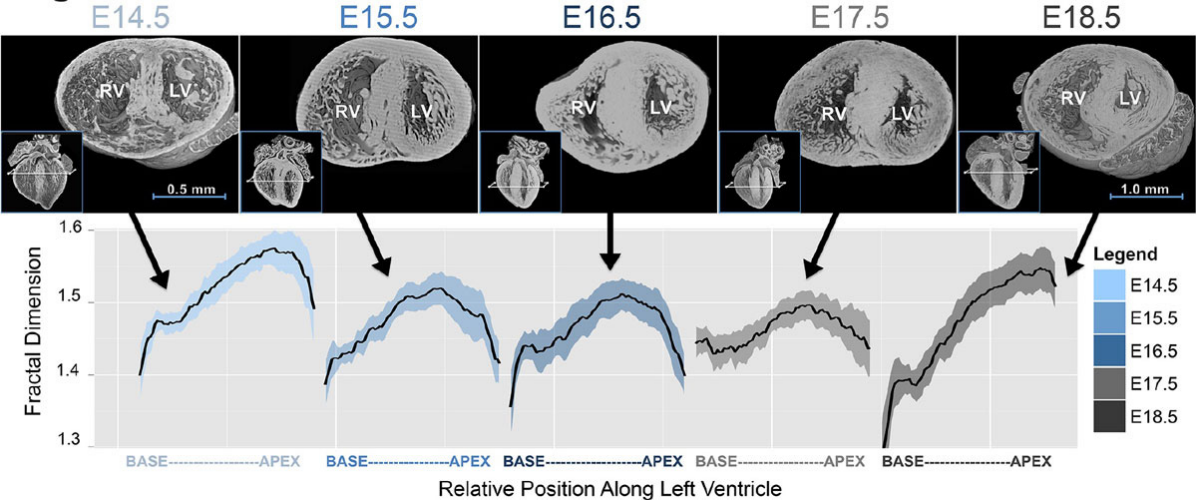
**Evolution of cardiac trabeculae in embryonic mice analyzed via fractal analysis of high-resolution (2-3 μm) episcopic microscopy datasets in complete registration**. This atlas of wild-type NIMR:Parkes mice provides the first detailed analyses of quantitative changes in the anatomical complexity of trabeculae during cardiac morphogenesis. The atlas was compiled from analyses of mice at ages: E14.5, n = 12; E15.5, n = 14; E16.5, n = 13; E17.5, n = 12 and E18.5, n = 12. Top section: Murine hearts in short axis with insert (bottom left) showing relative slice location along the LV. Scale bars in millimeters are included for E14.5 and E18.5. Bottom section: Black lines = mean fractal dimension; Coloured ribbons = 95% confidence intervals. E = embryonic day.

**Figure 2:**
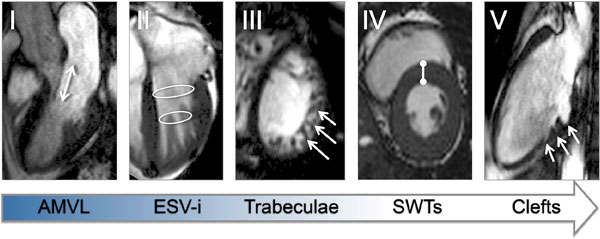
**A large population of G+LVH- carriers and matched healthy volunteers underwent detailed morphological assessment by CMR evaluating anterior mitral valve leaflet (AMVL) length in the 3-chamber view (method described by Maron et al.), diastolic and systolic wall thicknesses using a 16-segment approach, clefts, trabeculae and other routine CMR parameters**. Using conditional logistic regression and leave-one-out cross validation we developed a predictive model for gene carriership in HCM. By CMR and in this descending order of power (I to V), the combined presence of: an elongated AMVL (≥20.5 mm); decreased body surface area-indexed left ventricular end-systolic volume (ESV-i, ≤23.6 mls/m2); increased maximal apical fractal dimension (≥1.279); increased maximal septal systolic wall thickness (SWTs, ≥14.1 mm) and presence of clefts (≥1) predicted gene carriage in this case-control population with a model accuracy of 78%.

In mice a fractal atlas of trabecular development showed decreasing complexity across the basal LV(E14.5-18.5;p < 0.0001) while complexity in the mid/apical LV rose again just before birth(E17.5-18.5;p < 0.0001|Figure 1). Contrasting the UCL case-control populations 5 differences were found and borne out in the validation cohort. Across the combined HCMNet/UCL cohort these were:1)longer AMVL(22 ± 3 vs20 ± 3 mm|p < 0.0001), 2)increased maximal-apical trabecular complexity(1.242 ± 0.07 vs 1.196 ± 0.05|p < 0.0001), 3)increased maximal-septal systolic wall thickness(13 ± 3 vs 12 ± 2 mm|p = 0.02), 4)lower indexed-end-systolic LV volume(23 ± 6 vs 26 ± 7 mls/m2|p = 0.005), and 5)presence of clefts(35 vs 7%|p < 0.0001). Conditional logistic regression provided a model containing these parameters, which predicted gene carriage with a high level of accuracy(78%;Figure 2).

## Conclusions

Fractal analysis applied to microscopy or CMR permits robust trabecular quantification. Trabecular complexity is increased in HCM gene mutation carriers even in the absence of LVH. Myocardial architectural abnormalities are an early phenotype of sarcomere mutations; a pentad of cardiac architectural abnormalities by CMR exhibits potential for predicting genetic carriage in HCM.

## Funding

Dr Captur is funded by the University College London, UK (Graduate Research Scholarship) and by the European Union (Science and Technology Research Grant). Her work on HCMNet in Bethesda (NIH) and Boston (BWH) was funded by the UCL Charlotte and Yule Bogue Research Fellowship. Murine HREM Experiments are funded by the The Wellcome Trust (National Institute of Medical Research UK, Tim Mohun Group).

